# PILOT NUTRITION EDUCATION PROGRAM FOR VETERANS LIVING WITH DEMENTIA AND THEIR CAREGIVERS

**DOI:** 10.1093/geroni/igae098.2834

**Published:** 2024-12-31

**Authors:** Jamie Giffuni, Kelly Barton-Ort, Elizabeth Dennis, Odessa Addison

**Affiliations:** Baltimore and Loch Raven VAMC, Baltimore, Maryland, United States; Baltimore VA Medical Center, Baltimore, Maryland, United States; University of Maryland School of Medicine, Baltimore, Maryland, United States; University of Maryland School of Medicine, Baltimore, Maryland, United States

## Abstract

The prevalence of dementia is expected to increase as the Veteran population ages. Caring for a Veteran with dementia impacts the physical and mental health of the caregiver. Programs are needed to support the health and wellbeing of both Veteran and caregiver. Vets & Veggies is a whole health(y) aging program that provides free produce to Veterans visiting the Geriatric Wellness Center. This project describes the development of a novel, pilot nutrition program combining nutrition education with kitchen skill development and fresh produce access for Veterans living with dementia and their caregivers, Vets & Veggies for Caregivers (V&VC). One-hour in-person classes led by a registered dietitian with experience in geriatrics occurred weekly for 4 weeks. Topics included: Feeding tips for Caregivers/Caring for YOUR nutrition; MyPlate/Healthy Eating for Older Adults; the importance of protein intake; cooking with spices/low sodium cooking. Participants received free produce with recipes/tips for use, and adaptive kitchen equipment for safe preparation by the Veteran. Behavioral Risk Factor Surveillance System – Fruit and Vegetable Module, Caregiver Self Efficacy 8-Item Scale, Zarit Caregiving Burden 6-Item Survey, and the Mini Nutritional Assessment were completed by the caregiver pre and post-program. Two Veteran/caregiver dyads and one caregiver attended all classes with small positive trends in fruit and vegetable intake, decreased caregiving burden score, and improved nutrition assessment score. Participants enjoyed and looked forward to the classes. This pilot program demonstrated feasibility for developing and implementing a program designed to improve the whole health of Veterans living with dementia and their caregivers.

